# Factors related to compliance to anti-malarial drug combination: example of amodiaquine/sulphadoxine-pyrimethamine among children in rural Senegal

**DOI:** 10.1186/1475-2875-8-118

**Published:** 2009-06-04

**Authors:** Aurélia Souares, Richard Lalou, Ibra Sene, Diarietou Sow, Jean-Yves Le Hesran

**Affiliations:** 1 Institut de Recherche pour le Développement, UR10, Faculté de Pharmacie, 4 av de l'Observatoire, 75006 Paris, France; 2 Institut de Recherche pour le Développement, UMR151-LPED, Centre St Charles, Case 10, 13331 Marseille cedex 3, France; 3 District sanitaire de Mbour, Mbour, Sénégal; 4 District sanitaire de Thiès, Thiès, Sénégal

## Abstract

**Background:**

The introduction of new anti-malarial treatment that is effective, but more expensive, raises questions about whether the high level of effectiveness observed in clinical trials can be found in a context of family use. The objective of this study was to determine the factors related to adherence, when using the amodiaquine/sulphadoxine-pyrimethamine (AQ/SP) association, a transitory strategy before ACT implementation in Senegal.

**Methods:**

The study was conducted in five rural dispensaries. Children, between two and 10 years of age, who presented mild malaria were recruited at the time of the consultation and were prescribed AQ/SP. The child's primary caretaker was questioned at home on D3 about treatment compliance and factors that could have influenced his or her adherence to treatment. A logistic regression model was used for the analyses.

**Results:**

The study sample included 289 children. The adherence rate was 64.7%.   Two risks factors for non-adherence were identified: the children's age (8–10 years) (ORa = 3.07 [1.49–6.29]; p = 0.004); and the profession of the head of household (retailer/employee versus farmer) (ORa = 2.71 [1.34–5.48]; p = 0.006). Previously seeking care (ORa = 0.28 [0.105–0.736], p=0.001] satisfaction with received information (ORa =  0.45 [0.24–0.84]; p = 0.013), and the quality of history taking (ORa =  0.38 [0.21–0.69]; p = 0.001) were significantly associated with good compliance.

**Conclusion:**

The results of the study show the importance of information and communication between caregivers and health center staff. The experience gained from this therapeutic transition emphasizes the importance of information given to the patients at the time of the consultation and drug delivery in order to improve drug use and thus prevent the emergence of rapid drug resistance.

## Background

Early treatment with effective anti-malarial drugs is the main life-saving intervention for those at risk of malaria. However, drug treatment is threatened by growing resistance of *Plasmodium falciparum *to drugs that were once effective against the parasite. Chloroquine resistance has increased around the world, and this single drug treatment has become useless in most malaria-endemic areas. Resistance to sulphadoxine-pyrimethamine (SP) is also widespread and its use must soon be discontinued. Resistance to other anti-malarial drugs, such as amodiaquine, varies, but their useful therapeutic life also appears limited. Consequently, nearly all African countries have seen an unprecedented change in national drug policies in recent years. In compliance with WHO recommendations, countries have discontinued chloroquine, as it becomes ineffective, and now promote artemisin-based combination therapy (ACT), as first-line treatment for mild malaria.

Combining anti-malarial drugs with different modes of action has been shown to improve the efficacy of individual drugs and has been proposed as a means of delaying the spread of drug resistance and prolonging the life span of anti-malarial drugs [1]. ACT has been shown to increase cure rates [2]. However, for the successful deployment of ACT, several issues need to be addressed [3], including issues regarding patients' adherence, which has been poorly documented so far. Measuring baseline levels of adherence and identifying possible factors of non-adherence are important steps before the introduction of this new combination. Deciding on malaria drug policies remains a major challenge in several countries. Not only must the pharmacotherapeutic properties of the various available treatment options be considered, but issues such as end-users' ability to afford products, the level of financial support needed and expected practices in the use of the drugs must also be taken into account.

The patient's treatment adherence is determined by numerous factors, such as perceptions of the disease, perceptions of treatment (taste, cost, complexity of the schedule and side effects), patient factors, health staff factors and the relationship between patient and nurses [4,5]. Efforts to improve the use of anti-malarial treatments by providing the drugs through trained community health workers [6], training shopkeepers and wholesalers [7] and delivering prepackaged unit doses [8] or improved labeling [9], have all been shown to enhance the percentage of patients who receive and complete the recommended dose. Many of these interventions, which had a positive impact on treatment compliance, concern information provided to the patient and communication between physician, pharmacist and patient or caregiver. It was hypothesized that this plays an important role in increasing caregiver compliance to anti-malarial treatment in Africa, but its its impact relative to other factors is not currently known. Faced with an average chloroquine treatment failure of 25% [10], Senegal changed its national malaria policy from chloroquine (CQ) to amodiaquine/sulphadoxine-pyrimethamine (AQ/SP) in 2003. In 2006, they shifted to ACT, the non co-formulated combination amodiaquine/artesunate.

The introduction of this combination must be considered as a test before ACT implementation; it is an opportunity to assess health staff behaviour and public adherence to new combinations. One year after introducing the new treatment, a follow-up study was conducted to assess patient adherence and the factors that might be related to it.

## Methods

### Study area and population

The study was conducted in rural areas of the Thiès and Mbour departments, 70 km east and southeast of Dakar, respectively. The population, mainly of Sereer origin, lives traditionally on one food crop (millet), one cash crop (groundnuts) and livestock farming. To cope with the agricultural crisis, new activities arose such as temporary migration to urban centers, particularly the city of Mbour and tourist areas. Malaria is endemic with seasonal outbreaks. The annual rainy season is short (July–October), with malaria transmission mainly restricted from August to November. The average rainfall was 463 mm during the 1988–1996 period [11].

The study took place in 2004 during the rainy season. Five health centers were chosen that are representative of health care provision in rural Senegal (staff and technical means, population scattering around the health centres, recruitment area). Urban and touristic areas and health centres with special NGO's health interventions were avoided. A homogeneous geographic area representing 30 037 persons was defined. The selected health centres provided basic services to the study population: curative care, immunization, prenatal care, delivery and malnutrition management. The health structures are staffed by a nurse, a community health worker and birth attendants. There are no permanent physicians, midwives, laboratory facilities or emergency transportation. They were chosen for their sufficient patient attendance to ensure a daily minimum recruitment of ill children. The average number of patients attending per health center per day was 15 in October and slightly lower in September and November.

### Design and procedures

During the study period, all children between two and 10 years of age were identified by nurses during the consultation when they presented a presumptive mild malaria attack. All children were prescribed a single dose of SP on Day 0 (25 mg sulphadoxine/kg, 1.25 mg pyrimethamine/kg) and one daily dose of AQ for three days (10 mg/kg/day). A thick blood film was conducted to measure the parasitaemia at D0 to have biological confirmation of the diagnosis, but nurses did not know the results at the time of prescription. Each provider was given a chart showing the appropriate dosage by weight and age, defined by the Senegalese National Malaria Control Programme. No study team member was present at the health center. Each evening, identification forms and blood smears were received from the nurses. Children were included the day after the last treatment dose (D3), when mothers/guardians were visited at home to explain the study design and obtain their written consent. Apart from the study team, no one was aware of this visit prior to our arrival.

### Questionnaire

The questionnaire was addressed to caregivers and accompanying adults the day following end-of-treatment intake (D3) or during the following three days in case of absence (D4–D6). The individual questionnaire included nine sections with questions related to: respondents' socio-economic and demographic characteristics, history of febrile illness, consultation at the health center, compliance, child health status between D0 and the interview day, adherence to treatment, knowledge about malaria, source of information and attitudes towards health and drugs. If the caregiver was different from the accompanying adult, he/she was asked the latter questions from the section concerning the health center and consultation. The last section was addressed to children; they were asked about the drugs (intake, taste) and about their disease and recovery.

### Interviews

A sample of 28 families was selected according to the respective inclusion number in each health center. Structured interviews with caregivers took place in the home the week following the health center consultation and after the end of treatment. Structured interview guidelines were developed focusing on nine themes: disease history, consultation, new treatment, health center trust, locus of control, opinion regarding correspondence between prescription and what caregivers did, information on health and disease, use of written prescription, compliance and difficulties regarding drug intake.

### Definition of adherence

Two levels of adherence were defined. Firstly, those who took 80% of the prescribed dose of the two drugs were considered "adherent." Secondly, strict full adherence (SFA) for the prescribed dose described a patient who correctly followed the entire treatment including dose, duration and frequency. They were required to take the exact dose prescribed every day for the three days for AQ and the exact dose prescribed the first day for SP; a higher or a lower dose taken on any one day resulted in the patients' classification as "non-adherent."

### Data analysis

Questionnaire data were entered in Dbase. Sample characteristics and patient's classification were described as percentages and presented with 95% confidence intervals (CI). A logistic regression was used to identify factors related to compliance. All independent variables were tested individually (Chi^2^) and entered into the first model since they were associated with compliance below a 0.30 level of significance. A backward step-by-step binary logistic regression was used. Only statistically significant variables (p < 0.05) were kept in the final model. The STATA program was used (STATA^® ^8.0, 2003; Stata Corporation, College Station, Texas, USA).

Qualitative data were analysed using the thematic method. Interviews were transcribed in their entirety. From the transcribed conversations, patterns of experiences could be listed. All data that related to previously classified patterns were identified, and related patterns were catalogued into sub-themes.

## Results

### Population

Among the 304 patients aged two to ten years considered by the nurse to have mild malaria, 289 were included in the study. The remaining 15 were prescribed quinine by the nurse. One hundred and forty-four children had laboratory confirmed malaria (parasitaemia > 2500/μl) and 145 were diagnosed presumptively by the nurse (117 were negative and 28 had a parasitaemia < = 2500/μl). The median age for all recruited children was 5.4 years. The sex ratio (male/female) was 1:3. There were 14 caregivers (5.9%) who did not buy all the prescribed drugs: 2 (0.7%) did not buy AQ; 6 (2.1%) did not buy SP. The prescription's mean cost was approximately 0.88 € [0.34–2.52].

The main caregiver was the mother (69.5%) followed by the grand mother (13.2%) and the father (7.6%). For 17.3% of children, the accompanying adult at the health center was different from the caregiver. The median age of the caregiver was 36.7 years; 99.3% were Muslim. The predominant ethnic groups were: Sereer (60.5%), Wolof (27.3%) and Peul (11.8%). Within the study group, 83.1% had received no formal education, while 13.8% had been to primary school and 3.1% to secondary school. Some 93.4% were not able to read the written prescription.

### Patient's compliance

First, patients were considered adherent if they took 80% of the prescribed dose. Thus, 209 (72.3%) were adherent to AQ and 245 (84.8%) to SP. Hence, a total of 187 children (64.7%) were adherent to both drugs. Secondly, 39.4% of the children demonstrated strict full adherence for AQ and 60.9% for SP. Overall, 37.7% of the children strictly followed the prescription for the two drugs.

### Health facilities description

Patients were recruited in five health facilities. The impact of health facilities on treatment compliance was tested: one of the health facilities had no nurse at the time of the study and a community health worker was in charge of all consultations. He was being trained by an NGO to treat malaria with the new combinations within the framework of a test to assess the new drugs' implementation at the community level. It was noticed that compliance was better at this health center: 17/19 (89.5%) children were compliant, compared to 170/270 (62.3%) in the other health facilities (p < 0.05). Concerning strict full adherence, 68.4% of the caregivers administered treatment to the child following strict guidelines in this health facility, compared to 35.5% in other facilities (p < 0.05).

### Predictors of adherence

In bivariate analyses, adherence was not associated with biological confirmation of the disease (p = 0.434). Drug factors such as taste (p = 0.7), galenic syrup/tablets (p = 0.45) and side-effects (p = 0.53) were not significantly associated with adherence. Caregivers' factors, except those linked to the information they received, were not significantly associated with compliance: relationship to the child (p = 0.60), formal education (p = 0.58), adult literacy (p = 0.56), activities (p = 0.73) and earnings (p = 0.77).

The health center factors were not significantly linked to compliance: wait time (p = 0.47), health provider's status (p = 0.54) – nurse/community health worker – with the exception of the case of the particular health facility run by a health worker.

80% adherence (see Additional file 1) was significantly (p < 0.30) associated with information transmitted to caregivers about the discontinuation of chloroquine p = 0.28), the change in drugs (p = 0.12), the information source (p = 0.09) and satisfaction with the information received during consultation (p = 0.04). Only one factor was linked to attitudes: not needing to continue treatment if the child no longer appeared sick (p = 0.26). Two socio-economic factors, head of household employment (p = 0.16) and children's age (p = 0.01), were related to adherence. Factors related to history of febrile illness, the time between the first symptom and consultation (p = 0.08) and previously seeking health care from a traditional healer or a community health center during this period (p = 0.02), were also related to adherence. The attention the patient received in terms of the health care provider's attitude and discussion was linked to adherence (p = 0.002).

Strict full adherence was significantly associated with almost the same factors (see Additional file 2), except those related to children and caregiver, health status and difficulties in administering the treatment.

Logistic regression models were fitted to examine the influence of various predictive factors associated with 80% adherence and strict full adherence.

#### 80% adherence regression model

All the variables above were included at the start of the stepwise logistic regression. In the final model, two variables were risk factors for non-adherence: children eight to 10 years of age compared to young children from two to four years of age (OR = 3.07; p = 0.004); and head of household employment: retailer (OR = 2.71) or employee (OR = 2.54) versus farmer (p = 0.006). Three variables were protective factors for adherence: patients' perceived satisfaction with received information (OR = 0.45; p = 0.013); previously seeking health care before the health center consultation (OR = 0.278; p = 0.010) and correct nurses' interviews about illness and children (i.e.: nurse asked questions about history of febrile illness, symptoms and previously seeking health care) (OR = 0.382; p = 0.001). Some variables were not selected in the final model because they were not significant, but it was noted that difficulties in administering treatment (p = 0.149) and a long time-lapse between initial symptoms and consultation (p = 0.057) tended to affect adherence; additionally, improved adherence was observed when the health-care provider was the first source of health information (p = 0.171).

#### Strict full adherence

Two factors were significantly positively associated with strict full adherence: satisfaction about the received information (ORa = 0.546, p = 0.029) and another consultation before the consultation at the health center (ORa = 0.370; p = 0.009). Being not informed on reasons for discontinuing chloroquine tended to affect adherence (p = 0.125); correct nurses' interview (p = 0.083) and health-care provider as first source of health information (p = 0.100) were also observed as tending to improve compliance.

### Consultation and drug sale observations

The results from these observations highlighted the short duration of consultations, estimated between three and five minutes. The nurse briefly examined the children: he or she took temperature, weighed the child and sometimes did abdominal palpations and chest auscultation when the child presented abdominal pain or coughing. The nurse usually sat behind his or her desk and just spoke to caregivers without performing an examination or auscultation and only looked at the child's eyes to evaluate anemia. Most of the time, he or she prescribed without providing a diagnosis, and caregivers declared they were not provided information about drugs. On the other hand, most of the people were provided with information by the drug supplier; however, they felt that they had not been provided with enough information on drug intake. The average time spent on each explanation was < 1 minute. The supplier cut the packages and provided explanations while putting them in a bag. When patients were interviewed at the health center exit, most of them could not state the correct drug schedule and were mistaken about drugs and dosages.

### Caregivers' interview

#### Drug intake and accordance with the drug schedule

Almost all the caregivers said they followed up the drug schedule in spite of describing very unorthodox drug intake. Few mothers recognized they had made mistakes and did not consider stopping treatment before its completion as a mistake. From the caregiver's perspective, the goal is to cure the child's recovery, therefore, no longer administering drugs to a child who appears cured does not conflict with the drug schedule.

#### External locus of control

One mother told us: "*God gives the disease, the husband provides money and nurses have to cure the children*," expressing an opinion held by the majority. Locus of control is external when considering the disease and internal when considering the children's well being. Mothers explained their role to us as maintaining hygiene for children, their clothes and the home and providing good and healthy food. Concerning diseases, they all believed their role was to notice their child was ill, to ask the father or head of household for money and to take the child to the health center and then pray to God to cure their child. Mothers said they played a passive role in their children's cure, limited to accompanying the child to the health center. Only one of them told us about the leading role she played in managing first the drugs and then the child's cure.

#### New treatment

At the beginning of the rainy season, all the families stated they were not aware of the policy changes for malaria treatment. Despite its yellow color, amodiaquine was linked to chloroquine due to its bitterness and the shape of the tablets; it was even called "*yellow chloroquine*." Families were unaware of the concept of association – the use of two drugs to treat one disease. They did not associate SP with malaria; the caregivers mistook it for an antipyretic (aspirin or paracetamol) because of its similar characteristics (white color and size). At the end of the rainy season, people had heard about the new drug; they noticed children did not spit out the drugs and one even said, "*This year malaria is less dangerous with the new drugs*."

#### Use of written prescription

The written prescription is not used by the caregivers. They just keep it "*to prove they take care of their child*." On the one hand, 93.4% of the caregivers were unable to read it, on the other hand, the nurses just wrote the prescription for the drug provider. The only way to correctly administer the treatment to the child is to memorize it: "*You have to memorize everything when the pharmacist explains it to you; if you have any doubts, you will have to go back to the health center for more explanations*." Because of poor interpretation, the marks made on packages were a greater source of misunderstanding than a way to improve compliance.

## Discussion

This study aimed to analyse population adherence to new strategies to treat malaria and to provide useful information about factors related to compliance to these prescriptions. Few studies are available on anti-malarial combination adherence and too few of them broach factors that may have an impact on drug compliance. Most of these studies were intervention studies and evaluated the role of intervention – drug formulation and packaging [8,12] and community education [6,9] – on patient drug usage patterns. From these results, three groups of factors were found to have an impact on compliance to anti-malarial treatment.

### Patient characteristics

In this study, the population was quite homogeneous, preventing the use of socio-demographic factors that might have an impact on compliance. In particular, formal education and adult literacy were not associated with compliance, but almost all of the caregivers did not receive any formal education. In Zambia, children whose caretakers had received some education had a lower risk to be non-adherent [13]. In Uganda, Fogg also noticed education may have an effect on the patients' understanding of the instructions given in the clinic, the quality of the patients' relationship with the health care provider and the ability of the patient to interpret pictorial instructions [14].

The association between being an employee or retailer versus farmer and non-compliance seemed very surprising. It was hypothesized that a difference existed in exthe household's social organization in cases of farmer families. In this case, the caregiver is more frequently the grandmother (p < 0.001) and older (p < 0.001); different generations are living in the same compound, and the women share responsibilities. One may be responsible for the specific task of drug administration and children's health care. Conceivably, in this case, the woman has more time and knew more about administering treatment to children.

Compliance was better among young children (two to four years) than the oldest (from eight to 10 years). The oldest children were found to have more responsibilities in the household. Immediately after feeling better, they returned to their activities: going to school, farming and herding cattle. They were spending most of their time away from home, were under less parental or caregiver supervision and were becoming more responsible for their own care. They also had more freedom and could refuse to take the drugs or spit them out behind their caregivers' back. Hence, it was necessary to interview them to obtain complete information on drug intake. A few mothers told the interviewers that they had to ask the children directly about compliance. Children's own health beliefs must be considered, and patient education for children may be beneficial in improving treatment compliance [15].

### History and symptoms of febrile illness

In terms of the time-lapse between onset of symptoms and the health center consultation, it was noticed that those who had a previous consultation (traditional healer, community health center or another health center) were more compliant to treatment. Secondly, waiting for more than two days to take children to the health center was a risk factor of non-compliance.

This seems to be linked to the caregiver's anxiety; the more reactive people who go to the health center at the onset of symptoms or who have sought healthcare elsewhere were more worried about their children's health and subsequently more compliant to treatment. In France, comparison between emergency and non-emergency consultations also revealed that family's anxiety was correlated with improved compliance [16].

Caregivers stated they would not give the tablets to a child who no longer appeared sick. As one mother said, "*When your child is sick, all your thoughts are for him and you cannot forget the drugs; but when he is feeling better, your mind is on many other things, and it can happen that you forget*." White also noticed that people are less likely to take medicines once they feel they have recovered from an illness [2]. The same thing was observed in many compliance studies: compliance was better when symptoms were more apparent [17]. The same results were found for asthma; compliance is better for symptomatic treatment than disease-modifying drugs [18]. In developing countries, families state they do not have enough money to take all sick people to the health center; therefore keeping drugs to give to other ill people appears more economical [19]. In Ecuador, Yepez noticed among other factors that "*getting cured quickly*" did not foster compliance. Non-compliance was also associated with the drugs themselves: side effects and reluctance to take drugs [20].

### Information and relationships between patients and caregivers

Almost all the intervention studies reach a similar conclusion: that appropriate information and well-designed written material could have a positive impact on improving adherence and, with verbal consultation, are essential for enabling patients to make appropriate decisions about their medicine taking [6,9].

In this study, non-compliance seemed to be mainly linked to lack of information. Unclear or incomplete explanations were reported by 60% of the families interviewed as a cause of poor compliance in a survey made among asthmatic children [21,18]. Being satisfied with the received information during consultation had a positive impact on compliance. It was noticed that caregivers always received poor information on drug intake, and this information rarely corresponded to consultation guidelines. However, people who were satisfied completed their treatment at higher rates. This places emphasis on greater correspondence between caregivers' expectations and the actual information transmitted by health staff. Concerning the impact of patient satisfaction on behaviour, Ley points out: '*when a patient is not satisfied with the explanation or did not understand it, his or her dissatisfaction will create a barrier to treatment intake *'[22]. Quality of care in terms of medical history taking and counseling was sub-optimal. The quality of the nurses' interviews during consultation also appears to be a factor in improving compliance. This result refers to the overall quality of the relationship and lies beyond the actual questioning [23]. The discussion between nurse and patient plays an important role since the nurse can use it to improve the diagnosis; it gives the patient a feeling of being listened to as well. This analysis has been clearly described by Morin, adding that the relationship between doctor and patient plays an important role in the observed effects [5].

## Conclusion

Although improved anti-malarial drug usage faces significant logistical constraints, a number of simple, practical interventions – both at the community and clinic level – are effective: development of consultation guidelines, health staff training, health education in schools, patient counseling and illustrated instructions could be useful tools. Nevertheless given the multiplicity of factors contributing to poor adherence to medication, a multi-factorial approach is required; it cannot be solved through a single action. Improving compliance implies that patients and health staffs understand both how and why drugs should be taken correctly [24]: appropriate information is essential for enabling patients to make appropriate decisions about taking medicine.

Health officials will have to balance the need to make ACT widely accessible with concerns about minimizing their inappropriate use to give the few new alternatives available for the treatment of malaria a chance to succeed and to ensure treatment efficacy for the coming years by reducing therapeutic failures and subsequent chemoresistance.

## Competing interests

The authors declare that they have no competing interests.

## Authors' contributions

AS, RL, DS, IS and JYLH designed the study protocol; AS, RL, DS and IS carried out the study; AS, RL and JYLH analysed and interpreted the data; AS and RL drafted the manuscript. All authors revised the article critically and read and approved the final manuscript. AS and RL are guarantors of the paper.

## Supplementary Material

Additional file 1 **Supplementary table**. Factors associated with 80% adherence to antimalarial drug therapy. Click here for file

Additional file 2 **Supplementary table**. Factors associated with strict full adherence to antimalarial drug therapy. Click here for file
