# Supplementary material for: Factors related to compliance to anti-malarial drug combination: example of amodiaquine/sulphadoxine-pyrimethamine among children in rural Senegal
Source: Malar J. 2009 Jun 4;8:118. doi: 10.1186/1475-2875-8-118 (PMC2694834; doi:10.1186/1475-2875-8-118)
Supplement: Additional file 1 — Supplementary table. Factors associated with 80% adherence to antimalarial drug therapy. [file 1475-2875-8-118-S1.doc]

Additional file 1. Factors associated with 80 % adherence to antimalarial drug therapy

| **80% ADHERENCE** | | | | | | | | | |
| --- | --- | --- | --- | --- | --- | --- | --- | --- | --- |
| **Potential factor** | N | Adh. (%) | Non-adh. (%) | Crude OR | p value | Adj. OR | p value | [95 % CI] | |
| **Age** vs 2-4 years | 133 | 37,6 | 62,4 | 1 | 0.012 | 1 | 0.004 | __ | __ |
| 5-7 years | 79 | 43 | 57 | 0.92 | __ | 1.001 | __ | 0.493 | 2.033 |
| 8-10 years | 73 | 32,9 | 67,1 | 2.18 |  | 3.065 | **__** | 1.493 | 6.290 |
| **Health state** vs Improvement | 197 | 36.5 | 63.5 | 1 | 0.059 | __ | **__** | __ | __ |
| Symptoms persistance | 64 | 39.1 | 60.9 | 0.47 | __ | __ | **__** | __ | __ |
| Worsening | 28 | 42.9 | 57.1 | 0.72 | __ | __ | __ | __ | __ |
| **Received informations satisfaction** vs No | 193 | 33.2 | 66.8 | 1 | 0.039 | 1 | 0.013 | __ | __ |
| Yes | 96 | 46.9 | 53.1 | 0.57 | __ | 0.450 | **__** | 0.239 | 0.847 |
| **Source of information** vs Radio | 98 | 29.6 | 70.4 | 1 | 0.088 | 1 | 0.171 | __ | __ |
| Health care providers | 79 | 50.6 | 43.4 | 0.56 | __ | 0.612 | __ | 0.291 | 1.289 |
| Friends / Family | 64 | 34.4 | 65.6 | 1.06 | __ | 1.380 | **__** | 0.655 | 2.906 |
| Other | 47 | 38.3 | 31.7 | 0.50 | __ | 0.620 | ___ | 0.263 | 1.462 |
| **Correct nurses interview** vs No | 88 | 30.7 | 69.3 | 1 | 0.01 | 1 | 0.001 | 0.212 | 0.690 |
| Yes | 143 | 39.9 | 60.1 | 0.47 | __ | 0.382 | __ | __ | __ |
| **Previous health care resort** vs No | 251 | 35.5 | 64.5 | 1 | 0.02 | 1 | 0.010 | __ | __ |
| Yes | 38 | 52.6 | 47.4 | 0.37 | __ | 0.278 | **__** | 0.105 | 0.736 |
| **Household head employment** vs Farmer | 129 | 40.3 | 59.7 | 1 | 0.163 | 1 | 0.006 |  |  |
| Retailer | 80 | 36.2 | 63.8 | 1.60 | __ | 2.715 | **__** | 1.344 | 5.485 |
| Employee | 79 | 34.2 | 65.8 | 1.63 | __ | 2.538 | **__** | 1.265 | 5.091 |
| **Drug change knowledge** vs No | 223 | 36.3 | 63.7 | 1 | 0.121 | __ | **__** | __ | __ |
| Yes | 66 | 42.4 | 57.6 | 0.62 | __ | __ | __ | __ | __ |
| **Legnth before the consultation** vs < 2 days | 111 | 39.6 | 60.4 | 1 | 0.079 | 1 | 0.057 | __ | __ |
| 2-4 days | 145 | 36.5 | 63.5 | 1.53 | __ | 2.169 | **__** | 1.132 | 4.156 |
| > 5 days | 33 | 36.4 | 63.6 | 1.35 | __ | 1.321 | __ | 0.559 | 3.123 |
| **Stop treatment at end of symptoms** vs No | 58 | 31 | 69 | 1 | 0.26 | __ | __ | __ | __ |
| Yes | 230 | 39.6 | 60.4 | 0.71 | __ | __ | __ | __ | __ |
| **Chloroquine give up knowledge** vs Good information | 30 | 56.7 | 43.3 | 1 | 0.283 | __ | __ | __ | __ |
| Bad information | 23 | 43.5 | 56.5 | 1.44 | __ | __ | **__** | __ | __ |
| No information | 236 | 34.7 | 65.3 | 1.95 | __ | __ | __ | __ | __ |
| **Difficulties to administrate treatment** vs No | 256 | 39.1 | 60.9 | 1 | 0.289 | 1 | 0.149 | __ | __ |
| Yes | 30 | 30 | 70 | 1.51 | __ | 1.966 | __ | 0.785 | 4.926 |
