# Supplementary material for: Factors related to compliance to anti-malarial drug combination: example of amodiaquine/sulphadoxine-pyrimethamine among children in rural Senegal
Source: Malar J. 2009 Jun 4;8:118. doi: 10.1186/1475-2875-8-118 (PMC2694834; doi:10.1186/1475-2875-8-118)
Supplement: Additional file 2 — Supplementary table. Factors associated with strict full adherence to antimalarial drug therapy. [file 1475-2875-8-118-S2.doc]

Additional file 2. Factors associated with strict full adherence to antimalarial drug therapy

| **STRICT FULL ADHERENCE** | | | | | | | | | |
| --- | --- | --- | --- | --- | --- | --- | --- | --- | --- |
| **Potential factor** | N | Adh. (%) | Non-adh. (%) | Crude OR | p value | Adj. OR | p value | [95 % CI] | |
| **Received informations satisfaction** vs No | 193 | 33.2 | 66.8 | 1 | 0.023 | 1 | 0.029 | __ | __ |
| Yes | 96 | 46.9 | 53.1 | 0.56 | __ | 0.546 | __ | 0.317 | 0.939 |
| **Source of information** vs Radio | 98 | 29.6 | 70.4 | 1 | 0.034 | 1 | 0.100 | __ | __ |
| Health care providers | 79 | 50.6 | 43.4 | 0.41 | __ | 0.458 | __ | 0.237 | 0.884 |
| Friends / Family | 64 | 34.4 | 65.6 | 0.80 | __ | 0.877 | __ | 0.429 | 1.791 |
| Other | 47 | 38.3 | 31.7 | 0.68 | __ | 0.847 | __ | 0.390 | 1.842 |
| **Correct nurses interview** vs No | 157 | 33.1 | 66.9 | 1 | 0.134 | 1 | 0.083 | __ | __ |
| Yes | 74 | 43.2 | 56.8 | 0.65 | __ | 0.615 | __ | 0.355 | 1.066 |
| **Previous health care resort** vs No | 251 | 35.5 | 64.5 | 1 | 0.042 | 1 | 0.009 | __ | __ |
| Yes | 38 | 52.6 | 47.4 | 0.49 | __ | 0.370 | __ | 0.175 | 0.784 |
| **Legnth before the consultation** vs < 2 days | 111 | 39.6 | 60.4 | 1 | 0.297 | __ | __ | __ | __ |
| 2-4 days | 145 | 36.5 | 63.5 | 1.14 | __ | __ | __ | __ | __ |
| > 5 days | 33 | 36.4 | 63.6 | 1.15 | __ | __ | __ | __ | __ |
| **Stop treatment at end of symptoms** vs No | 58 | 31 | 69 | 1 | 0.231 | __ | __ | __ | __ |
| Yes | 230 | 39.6 | 60.4 | 0.69 | __ | __ | __ | __ | __ |
| **Chloroquine give up knowledge** vs Good information | 30 | 56.7 | 43.3 | 1 | 0.055 | 1 | 0.125 | __ | __ |
| Bad information | 23 | 43.5 | 56.5 | 1.70 | __ | 1.513 | __ | 0.476 | 4.814 |
| No information | 236 | 34.7 | 65.3 | 2.46 | __ | 2.293 | __ | 0.986 | 5.332 |
